# Identification of Immune Infiltration-Associated CC Motif Chemokine Ligands as Biomarkers and Targets for Colorectal Cancer Prevention and Immunotherapy

**DOI:** 10.3390/ijms26020625

**Published:** 2025-01-13

**Authors:** Minghao Liu, Teng Wang, Mingyang Li

**Affiliations:** Centre of Biomedical Systems and Informatics, ZJU-UoE Institute, School of Medicine, International Campus, Zhejiang University, Haining 314400, China; minghao1.24@intl.zju.edu.cn (M.L.); tengw.21@intl.zju.edu.cn (T.W.)

**Keywords:** bioinformatics analysis, CC chemokine, colorectal cancer, immune infiltration, therapeutic target, biomarker

## Abstract

Colorectal cancer (CRC) is the third most common cancer globally, with limited effective biomarkers and sensitive therapeutic targets. An increasing number of studies have highlighted the critical role of tumor microenvironment (TME) imbalances, particularly immune escape due to impaired chemokine-mediated trafficking, in tumorigenesis and progression. Notably, CC chemokines (CCLs) have been shown to either promote or inhibit angiogenesis, metastasis, and immune responses in tumors, thereby influencing cancer development and patient outcomes. However, the diagnostic and prognostic significance of CCLs in CRC remains unclear. In this study, multiple online tools for bioinformatics analyses were utilized. The findings revealed that the mRNA expression levels of CCL3, CCL4, and CCL26 were significantly elevated in CRC tissues compared to normal tissues, whereas CCL2, CCL5, CCL11, CCL21, and CCL28 mRNA levels were markedly downregulated. Additionally, dysregulation of CCL4, CCL5, and CCL21 was strongly associated with clinical staging, and elevated levels of CCL4, CCL11, and CCL28 were linked to significantly prolonged survival in CRC patients. Functional enrichment analysis indicated that the cellular roles of CCLs were predominantly associated with the chemokine, Wnt, and Toll-like receptor signaling pathways, as well as protein kinase activity. Furthermore, transcriptional regulation of most CCLs involved RELA and NFKB1. Key downstream targets included members of the SRC family of tyrosine kinases (HCK, LYN, and LCK), serine/threonine kinases (ATR and ATM), and others such as CSNK1G2, NEK2, and CDK2. Moreover, CCLs (CCL2, CCL3, CCL4, CCL5, CCL11, CCL21, and CCL28) exhibited strong correlations with major infiltration-related immune cells, including B cells, CD8^+^ T cells, CD4^+^ T cells, macrophages, neutrophils, and dendritic cells. In conclusion, our study provides novel insights into the potential utility of CCLs as biomarkers and therapeutic targets for CRC prevention and immunotherapy.

## 1. Introduction

Colorectal cancer (CRC) is the third most common cancer in Western countries and a leading cause of death worldwide [1]. Most CRC patients are diagnosed at advanced stages due to the absence of sensitive biomarkers [2]. Even when diagnosed early, the prognosis remains poor, largely due to a lack of effective therapeutic targets in clinical practice [3]. Recent research into the tumor microenvironment, particularly immune infiltration, has highlighted immunotherapy as an emerging approach to CRC treatment, supplementing traditional radiotherapy and chemotherapy [4]. While some immune-related markers, such as immune checkpoints [5], have been investigated, there remains a need to identify additional biomarkers. Chemokines, which play a critical role in cancer immune evasion, are closely associated with the onset and progression of cancers, including CRC [6,7]. Therefore, exploring the diagnostic and prognostic potential of chemokines is of significant interest.

The CC subfamily of chemokines, known as CC motif chemokine ligands (CCLs) [8], comprises a group of chemotactic cytokines characterized by an N-terminal CC domain, with their numerical designations based on their order of discovery [9]. In humans, 27 CCLs have been identified, although CCL9 and CCL10 are the same, and commonly studied members include CCL1, CCL2, CCL3, CCL4, CCL5, CCL6, CCL11, CCL21, CCL26, and CCL28. These chemokines typically facilitate the body’s immune response by attracting immune cells to combat foreign agents such as invading pathogens [10]. Notably, recent studies have revealed that the dual roles of CCLs, both promoting and inhibiting cancer, are pivotal in tumorigenesis and progression, particularly in CRC [11]. Among these, CCL2 has been the most extensively studied, with evidence suggesting its role in inducing drug resistance in breast and CRC cells, underscoring its potential as a diagnostic biomarker and therapeutic target [12,13]. However, the functions of other CCLs in cancer diagnosis and treatment remain poorly understood.

Similar to other chemokines, CCLs and their receptors play a critical role in regulating immune system cells [14]. Among these, CCR2, which is downregulated by CCL2 in cancer cells, has been linked to several alterations, including enhanced immune responses, upregulated MHC class I expression, and reduced expression of the checkpoint regulator PD-L1 [15]. Notably, blocking β-Catenin can induce CCL28, which suppresses gastric cancer progression by inhibiting immune cell infiltration [15]. Additionally, the p65/STAT3-CSN5-PD-L1 pathway has been identified as a crucial mechanism through which macrophage-derived CCL5 facilitates immune evasion in colorectal cancer cells [7]. These findings underscore that the functional network of CCLs is intricately connected to their upstream and downstream factors, as well as their regulatory pathways.

This study conducted a comprehensive and systematic bioinformatics analysis of CCLs in CRC. First, the differential expression of these CCLs was identified in tumor tissues. Next, their potential as indicators for predicting CRC progression and prognosis was evaluated by analyzing their correlations with pathological stages and patient outcomes. The functional network of CCLs was then delineated by examining their protein–protein interaction, signaling pathways, upstream transcription factors, and downstream kinase targets. Furthermore, the effects of CCLs on immune cell infiltration in CRC were explored. In summary, these selected CCLs were proposed as potential biomarkers and therapeutic targets for CRC prevention and immunotherapy, based on their specific roles in CRC.

## 2. Results

### 2.1. Dysregulation of CCLs in CRC Samples

**Figure 1:**
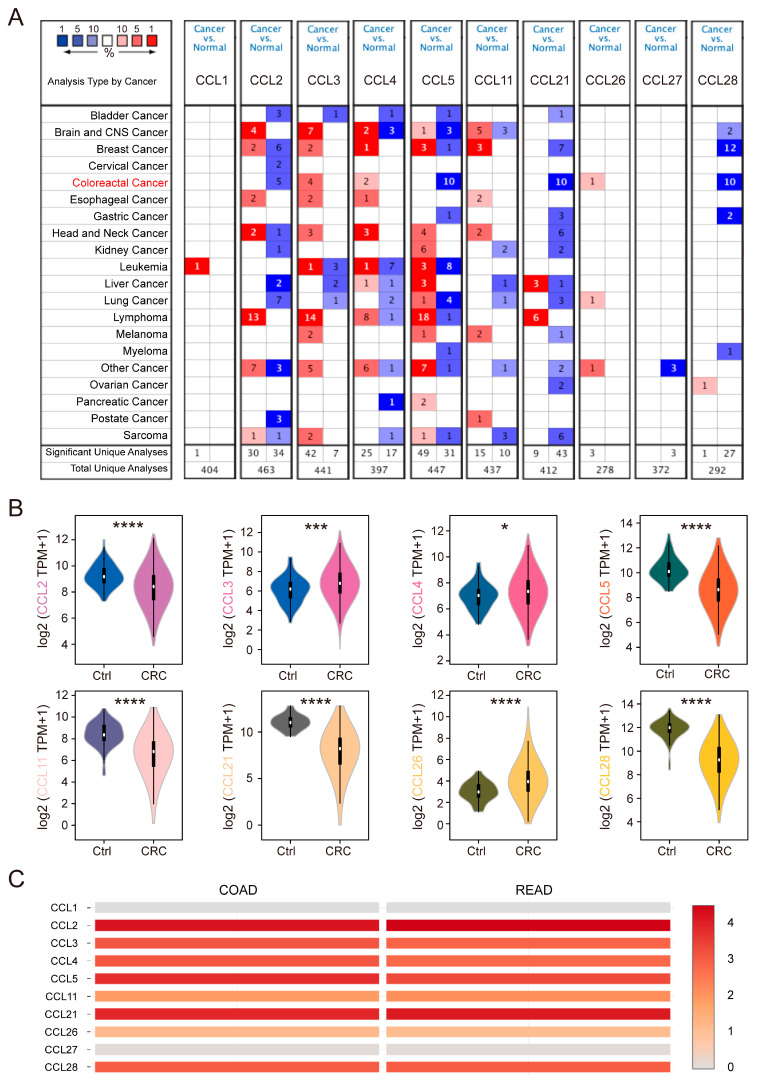
mRNA levels of CCLs in CRC. (**A**) The numbers of datasets with significant CCL mRNA overexpression (red) or downregulated expression (blue) based on ONCOMINE. (**B**) mRNA levels of CCLs in CRC compared with normal tissues from the TCGA database; data were expressed as medians (*, *p* < 0.05; ***, *p* < 0.001; ****, *p* < 0.0001). (**C**) The relative level of CCLs in CRC using GEPIA.

The ONCOMINE database was accessed to analyze the expression patterns of ten commonly dysregulated CCLs (CCL1, CCL2, CCL3, CCL4, CCL5, CCL11, CCL21, CCL26, CCL27, and CCL28) in CRC. Compared with controls, the mRNA levels of CCL3, CCL4, and CCL26 were significantly elevated in CRC datasets, while the mRNA levels of CCL2, CCL5, CCL21, and CCL28 were markedly reduced (Figure 1A). Representative datasets are summarized in Table 1. Notably, a significant downregulation of CCL2 was observed in rectal adenoma (a benign lesion) with a fold change (FC) of −3.318 and a *p*-value of 2.82 × 10^−4^. Conversely, CCL3 (FC = 3.319, *p* = 4.30 × 10^−7^) and CCL4 (FC = 2.405, *p* = 1.76 × 10^−6^) were significantly upregulated in CRC. Dysregulation of other CCLs, including CCL5 (FC = −8.903, *p* = 5.76 × 10^−4^), CCL11 (FC = −7.038, *p* = 9.50 × 10^−8^), and CCL21 (FC = 5.276, *p* = 1.15 × 10^−7^), was also identified using TCGA data. Additionally, in the dataset from Hong Y et al. [16], the expression of CCL26 was notably lower in CRC tissues compared to normal tissues (FC = 5.276, *p* = 1.15 × 10^−7^).

To validate these findings, mRNA levels of the selected CCLs were determined using TCGA data and the results were visualized and analyzed using violin plots. As anticipated, the expression patterns were consistent with our previous observations, showing significant differences between CRC and normal samples (Figure 1B). Further analysis of relative CCL expression levels in CRC tissues revealed that CCL2 exhibited the highest expression among the selected CCLs (Figure 1C). However, CCL1 and CCL27 were excluded from further analysis due to their negligible expression levels in CRC (Figure 1C). These findings indicate that most CCLs play a role in the regulation of CRC tumorigenesis.

### 2.2. Correlation of CCLs with Pathological Stage and Patient Outcome of Patients with CRC

**Figure 2:**
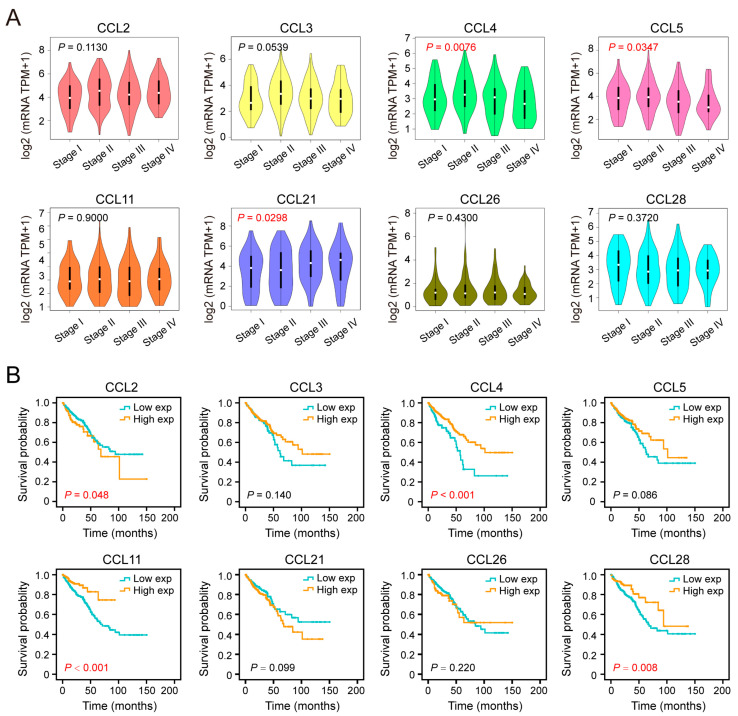
Correlation of CCLs with pathological stage and clinical outcomes in CRC. (**A**) The expression differences in CCLs among various pathological stages of CRC patients based on GEPIA. (**B**) The prognostic value of these indicators in CRC patients was assessed by drawing the overall survival curve on data from HPA.

The clinical significance of abnormal CCL expression is noteworthy. To explore this further, the relationship between abnormal CCL expression and the clinical stage and survival outcomes of CRC patients was analyzed. Specifically, the expression levels of CCLs across different pathological stages of CRC were compared using the GEPIA database. The analysis revealed that CCL4 (*p* = 0.0076) and CCL5 (*p* = 0.0347) expression levels tended to decrease as the tumor progressed (Figure 2A). In contrast, CCL21 expression (*p* = 0.0298) was elevated in advanced CRC (Figure 2A). However, other CCLs, including CCL2, CCL3, CCL11, CCL26, and CCL28, did not exhibit significant changes in expression during tumor progression.

Furthermore, survival analysis using the Kaplan–Meier method and log-rank test demonstrated that high CCL2 expression was associated with shorter survival times in CRC patients (*p* = 0.048), identifying it as a potential risk factor for poor prognosis (Figure 2B). Conversely, elevated levels of CCL4 (*p* < 0.001), CCL11 (*p* < 0.001), and CCL28 (*p* = 0.008) were linked to improved clinical outcomes in CRC patients (Figure 2B). The expression of CCL3, CCL5, CCL21, and CCL26 showed no significant association with survival probabilities. These findings suggest that specific CCLs play critical roles in tumor progression and may serve as potential prognostic markers in CRC.

### 2.3. Genetic Alteration, Co-Expression, and Protein–Protein Interactions of CCLs in CRC Cells

**Figure 3:**
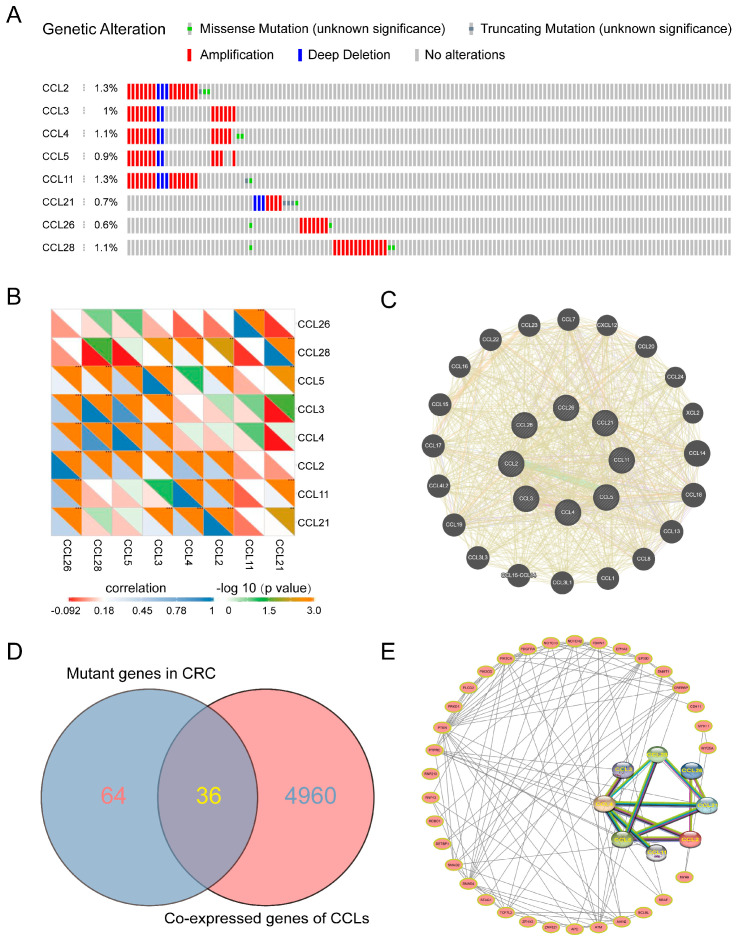
Genetic alteration, mutual expression, and protein–protein interaction of CCLs in CRC. (**A**) Summary of alterations in CCLs in CRC based on samples from cBioPortal (n = 1510). (**B**) Heat map showing the correlations among these CCLs in CRC (*, *p* < 0.05; **, *p* < 0.01; ***, *p* < 0.001). (**C**) Gene–gene interaction network of different expressed CCLs using GeneMANIA. (**D**) Venn diagram gathering the common genes that were simultaneously mutated and co-expressed with CCLs. (**E**) Protein–protein interaction network of these CCLs and the expression products of these co-expressed mutated genes by searching STRING.

To investigate the genetic alterations and molecular characteristics of CCLs in CRC, CRC samples from TCGA (n = 1510) were analyzed. As shown in Figure 3A, the alteration frequencies of CCL genes (CCL2, CCL3, CCL4, CCL5, CCL11, CCL21, CCL26, and CCL28) in CRC were 1.3%, 1.0%, 0.9%, 1.3%, 0.7%, 0.6%, 1.1%, and 1.1%, respectively. Notably, all eight CCLs exhibited abnormal amplifications, while missense mutations were observed in all except CCL3 and CCL5. In contrast, deep deletions were absent in CCL26 and CCL28.

The co-expression relationships of mRNAs among these dysregulated CCLs were further explored. The analysis revealed significant correlations between several pairs, including CCL2 and CCL6, CCL3 and CCL28, CCL4 and CCL5, CCL21 and CCL28, CCL1 and CCL26, CCL4 and CCL11, and CCL2 and CCL21 (Figure 3B). Using GeneMANIA, a protein domain similarity of 44.93% among the selected CCLs and genetic interactions of 18.38% between CCL2 and CCL5 was identified (Figure 3C). Additionally, the genes co-expressed with CCLs and those mutated in CRC were intersected (Figure 3D). This analysis enabled us to map the network of protein–protein interactions involving these pivotal CCLs (Figure 3E). Collectively, these findings highlight the intricate relationships among the eight CCLs and the genes interacting with them, shedding light on their potential roles in CRC pathogenesis.

### 2.4. Cellular Function of the CCLs Performing in CRC

**Figure 4:**
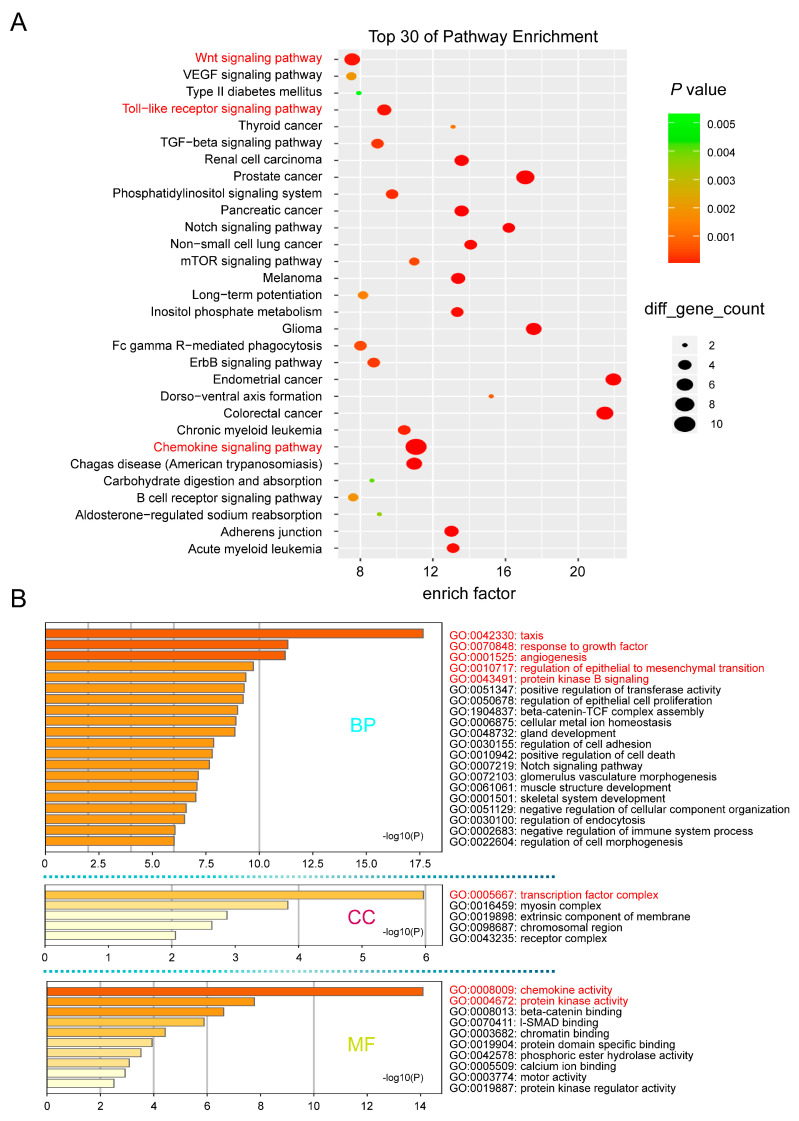
The enrichment analysis of CCLs and co-expressed mutated genes in CRC. (**A**) Bubble diagram of KEGG-enriched terms using Shbio. (**B**) Bar plot of GO enrichment in cellular component terms, biological process terms, and molecular function terms based on Metascape.

As established in the literature, dysregulated CCLs play critical roles in pathways associated with CRC tumorigenesis and progression, including the chemokine signaling pathway, Wnt signaling pathway, and Toll-like receptor signaling pathway [22,23]. These pathways, as revealed by KEGG analyses, are illustrated in Figure 4A. GO analysis further indicated that these CCLs are involved in tumor-related biological processes such as taxis, response to growth factors, angiogenesis, regulation of epithelial-to-mesenchymal transition, and protein kinase B signaling (Figure 4B).

In the CC category, the transcription factor complex emerged as the most enriched item (Figure 4B), suggesting that CCLs are predominantly regulated by transcription factors. Notably, the MF category revealed significant enrichment not only in chemokine activity but also in protein kinase activity (Figure 4B), both of which are essential for maintaining tumor characteristics in CRC. These findings highlight the potential involvement of transcription factors and protein kinases in CCL-related regulatory networks.

### 2.5. Transcription Factors and Kinase Target of CCLs in CRC

To further elucidate the regulatory networks of CCLs, TRRUST and LinkedOmics were utilized to identify relevant transcription factors and kinase targets. Our analysis identified CCL2, CCL3, CCL4, CCL5, CCL11, CCL21, and CCL26 as key CCLs in TRRUST. Among these, RELA and NFKB1 were confirmed as shared transcription factors for CCL2, CCL3, CCL4, CCL5, and CCL11, consistent with previous studies (Table 2) [24,25,26,27,28]. Additional upstream regulators included IRF3, REL, STAT6, SPI1, STAT1, STAT3, JUN, and SP1 (Table 2), further underscoring the complexity of the transcriptional regulation of CCLs. Kinase targets for these CCLs were also identified, with the top two targets listed in Table 3. For instance, HCK and PIK3CD were downstream targets of CCL2, while LYN and HCK were regulated by CCL3. Both LCK and LYN were identified as critical kinases in the CCL2 and CXCL3 networks. Similarly, LYN and CSNK1G2 were validated as effective targets of CCL11. The primary downstream kinases of CCL21 were NEK2 and CSNK1G2. Furthermore, LYN and ATR were significant components of the CCL26 and CCL28 kinase–target networks, along with ATM and CDK2.

### 2.6. Correlation of CCLs with Immune Cell Infiltration in CRC

**Figure 5:**
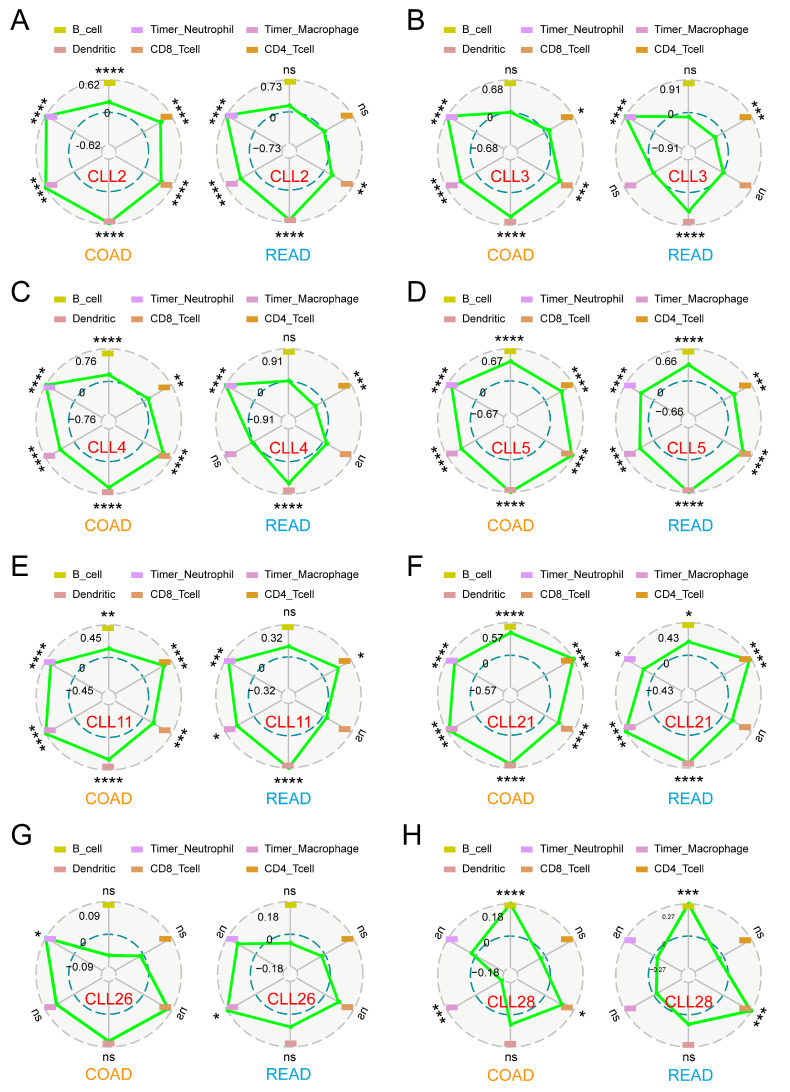
The correlation between CCLs and immune cell infiltration in CRC. The correlation between the abundance of immune cells and the expression of (**A**) CCL1, (**B**) CCL2, (**C**) CCL3, (**D**) CCL4, (**E**) CCL5, (**F**) CCL11, (**G**) CCL21, and (**H**) CCL28 in CRC using Sanger Box (ns, *p* > 0.05; *, *p* < 0.05; ** *p* < 0.01; ***, *p* < 0.001; ****, *p* < 0.0001). “TIMER_Neutrophil” and “TIMER_Macrophage” are metrics of immune infiltration correlation.

CCLs play a crucial role in modulating the tumor microenvironment, particularly by influencing immune cell infiltration, including B lymphocytes, Timer_Neutrophils, Timer_Macrophages, dendritic cells, CD8^+^ T cells, and CD4-positive cells. This interaction significantly impacts tumor progression and prognosis in CRC [9]. To investigate this further, a comprehensive analysis was conducted using the Sangerbox platform to explore the correlation between CCL expression and immune cell infiltration in CRC. The analysis revealed a consistent positive correlation between CCL2 and Timer_Neutrophils, Timer_Macrophages, dendritic cells, and CD8^+^ T cells in CRC (Cor > 0, *p* < 0.05; Figure 5A). Similarly, CCL3 expression showed a positive association with Timer_Neutrophils, dendritic cells, and CD4-positive cells (Cor > 0, *p* < 0.05; Figure 5B). Likewise, CCL4 exhibited a similar positive correlation with these immune cells (Cor > 0, *p* < 0.05; Figure 5C). Notably, CCL5 was significantly associated with the infiltration of all six immune cell types analyzed (Cor > 0, *p* < 0.05; Figure 5D). CCL11 expression correlated positively with four immune cell types, excluding B lymphocytes and CD8^+^ T cells (Cor > 0, *p* < 0.05; Figure 5E). Additionally, CCL21 levels were positively associated with five immune cell types, including B lymphocytes, Timer_Neutrophils, Timer_Macrophages, dendritic cells, and CD4-positive cells (Cor > 0, *p* < 0.05; Figure 5F). In contrast, no significant correlation was observed between CCL26 and any of the immune cells (*p* > 0.05; Figure 5G). Finally, dysregulated CCL28 expression was closely linked to B lymphocytes and CD8^+^ T cells (Cor > 0, *p* < 0.05; Figure 5H).

To further evaluate the prognostic implications, a multivariate Cox regression model, incorporating the eight dysregulated CCLs and six immune cell types, was constructed. As shown in Table 4, CCL11 and CD8^+^ T cells emerged as independent protective factors for CRC prognosis (HR < 1, *p* > 0.05). These findings confirm that several CCLs (CCL2, CCL3, CCL4, CCL5, CCL11, CCL21, and CCL28) are involved in regulating immune cell infiltration, with the exception of CCL26. Moreover, CCL11 shows potential as an independent prognostic indicator for CRC.

## 3. Discussion

Currently, despite the widespread adoption of first-line surgical and chemotherapy regimens, there has been no significant improvement in patient survival rates. This underscores the urgent need to identify more sensitive therapeutic targets for CRC treatment [29]. Given the intricate mechanisms underlying tumor development, the investigation of tumor microenvironment imbalances, particularly immune escape, may offer a promising avenue for new interventions [30]. In this study, dysregulated CCLs were identified as potential targets for CRC immunotherapy.

CCLs, a subfamily of soluble cytokines, function as chemoattractants that guide the migration of various cells, particularly immune cells [31]. Initially recognized as inflammatory mediators, CCLs have since been implicated in cell proliferation, differentiation, and survival [32]. Their dynamic expression patterns make them pivotal in shaping the tumor microenvironment. Emerging evidence highlights the critical role of CCLs in tumor cell proliferation, apoptosis, and metastasis, primarily through their involvement in immune escape mechanisms [33,34,35]. For instance, studies have demonstrated that elevated CCL2 expression correlates with increased infiltration of CD163^+^ macrophages, and patients exhibiting high CCL2 levels experience shorter progression-free survival [12]. Similarly, CCL21 has been shown to enhance migration, invasion, tumor sphere formation, and colony formation in oral squamous cell carcinoma [36]. Notably, CCL5 has been identified as a key player in tumor initiation and progression by facilitating immune evasion [37]. Despite these findings, the diagnostic and therapeutic potential of CCLs in CRC remains inadequately explored. Further research is urgently needed to elucidate the functional networks and clinical relevance of CCLs in CRC.

The dysregulation of CCLs and their correlation with pathological stages and clinical outcomes in CRC were initially verified. Our findings demonstrated that high expression levels of CCL4, CCL11, and CCL28 are associated with improved patient outcomes, positioning them as potential prognostic markers. Conversely, changes in the expression of CCL2, CCL5, and CCL21 were linked to tumor stages, influencing tumor growth and metastasis by modulating immune cell activity, thereby highlighting their value as therapeutic targets. Eight genes were identified as differentially expressed in CRC compared to controls. Among these, CCL3, CCL4, and CCL26 were upregulated in CRC, while CCL2, CCL5, CCL21, and CCL28 were downregulated. Furthermore, CCL4 and CCL5 expression decreased as CRC progressed, whereas CCL21 levels increased in advanced tumors. Notably, CRC patients with high CCL2 expression exhibited shorter survival times, whereas elevated levels of CCL4, CCL11, and CCL28 were linked to improved prognoses. These findings underscore the critical roles of these CCLs in tumor progression and their potential utility in assessing CRC prognosis. Similarly, prior studies have verified that high CCL2 expression correlates with poor CRC prognosis [13], while abnormally elevated CCL21 expression is closely associated with poor outcomes in extrahepatic cholangiocarcinoma [38]. However, the comprehensive exploration of the roles of all CCLs in cancer progression and prognosis, particularly in CRC, remains limited.

Tumorigenesis and CRC progression are complex and multifaceted processes, with genomic heterogeneity playing a pivotal role [39]. To investigate the differential expression of multiple CCLs in CRC, their genetic alterations were analyzed. As expected, frequent abnormalities such as amplifications, missense mutations, and deep deletions were observed in these CCLs. Additionally, dysregulated CCLs were closely correlated with one another, suggesting that these cytokines synergistically modulate CRC tumorigenesis and progression. Similar findings have been reported for the CXC chemokine subfamily [40].

To further elucidate the cellular functions of the selected CCLs in CRC, GO and KEGG analyses were performed. These analyses revealed that the CCLs are primarily enriched in pathways such as the chemokine signaling pathway, Wnt signaling pathway, and Toll-like receptor signaling pathway [41,42,43]. They are also involved in processes such as response to growth factors, angiogenesis, regulation of epithelial-to-mesenchymal transition, and protein kinase B signaling, all of which play critical roles in CRC tumorigenesis and progression [44,45,46]. Additionally, transcription factor complexes and protein kinase activity were identified as closely associated with these CCLs. These findings provide robust evidence for the emerging roles of CCLs in cellular functions, further supporting their potential as therapeutic drug targets.

Moreover, the functional network of chemokines was validated by analyzing the transcription factors and enzyme targets associated with chemokines. Our findings confirmed that RELA and NFKB1, previously reported in the literature, are common transcription factors for CCL2, CCL3, CCL4, CCL5, and CCL11 [24,25,26,27,28]. This study provides a fresh perspective on the transcriptional regulatory network of CCLs. Additionally, the SRC family of tyrosine kinases (HCK, LYN, and LCK) and the serine/threonine kinases (ATR and ATM) emerged as major potential targets of the differentially expressed CCLs, introducing a novel CCL–kinase axis. Notably, these kinases have been implicated in the regulation of innate immune responses and DNA damage repair mechanisms [47,48]. Thus, the selected CCLs may influence DNA repair and cytophagy by modulating these kinases in CRC.

Recent studies have highlighted the pivotal role of immune cell infiltration in tumor progression, recurrence, and response to immunotherapy, as well as its utility as a predictor of clinical outcomes [49]. For instance, an immune cell infiltration-based signature has been developed for overall survival prediction and treatment guidance in breast cancer patients [50]. Similarly, tumor immune infiltration-associated lncRNAs have been identified to improve prognosis and immunotherapy response in non-small-cell lung cancer [51]. In this study, significant associations between CCL expression and six types of immune cells, including B lymphocytes, Timer_Neutrophils, Timer_Macrophages, dendritic cells, CD8^+^ T cells, and/or CD4^+^ T cells, were observed. Among these, CCL11 and CD8^+^ T cells were identified as independent protective factors for CRC prognosis. These findings suggest that the level of immune infiltration is a critical factor in the involvement of CCLs in CRC progression.

However, our study has some limitations. While transcriptional analysis provides insights into immune status, it does not capture global changes comprehensively. Furthermore, the inclusion of an additional validation cohort would strengthen our findings. Refinement of both in vitro and in vivo experiments is also necessary to enhance the reliability and robustness of our conclusions.

In conclusion, dysregulated CCLs are involved in tumorigenesis, clinical outcomes, and immune cell infiltration. Our work contributes to a deeper understanding of the role of chemokines in immune infiltration, offering valuable insights for the development of effective biomarkers and therapeutic targets for CRC prevention and immunotherapy.

## 4. Materials and Methods

### 4.1. Database Selection and Search Strategy

Rationale for Database Selection: To identify CCLs associated with immune infiltration in colorectal cancer (CRC) as potential biomarkers and therapeutic targets, we selected databases that provide comprehensive information on gene expression, genetic alterations, protein interactions, and immune cell infiltration specific to CRC and CCLs. The databases selected—ONCOMINE, GEPIA, cBioPortal, UALCAN, GeneMANIA, Metascape, TRRUST, LinkedOmics, TIMER, and SangerBox—offer robust datasets essential for exploring these factors.

Search Strategy: A systematic search of the databases was conducted using keywords such as “CCL”, “colorectal cancer”, and “immune infiltration”, with a focus on CCL expression, genetic variations, and their correlation with immune cell infiltration in CRC. Databases like ONCOMINE and GEPIA were used to examine gene expression differences, cBioPortal for genetic alterations, and GeneMANIA and STRING for interaction networks. TIMER and SangerBox were utilized to analyze correlations between CCLs and immune cell infiltration.

Inclusion and Exclusion Criteria: Studies and datasets were included if they provided data on CCL expression, genetic alterations, or immune cell infiltration in CRC. We excluded studies that lacked relevance, had insufficient data on immune cell infiltration, or lacked robust statistical support.

### 4.2. ONCOMINE

The ONCOMINE platform (https://www.oncomine.org/) is a cancer microarray database and web-based data-mining tool designed to facilitate discoveries from genome-wide expression analyses. It provides gene expression signatures, clusters, and gene set modules to support research into cancer biology [52]. We utilize this database for identifying CCLs that are differentially expressed in colorectal cancer (CRC). In our study, the expression of crucial CCLs was compared between cancer and normal tissues mainly in CRC via the “Gene Summary View” and “Dataset View” interfaces, respectively, and the threshold values of the difference in CCL expression were obtained from Student’s *t*-tests, which were set with the following parameters: |Log2 FC| > 1, *p* ≤ 0.05, and gene rank in the top 10%.

### 4.3. GEPIA

GEPIA (http://gepia.cancer-pku.cn/), a web server for cancer and normal gene expression profiling and interactive analysis based on TCGA and the GTEx projects [53], was used in this study to compare the mRNA levels among the selected ten CCLs and their correlations with a pathological stage in order to correlate CCL expression with CRC stages and assess their role in tumor progression. Specifically, multiple gene expression differences in CCLs in CRC were first displayed as an interactive heat map with the “Multiple Gene Comparison” module on this platform, via the “COAD” (colon adenocarcinoma) and “READ” (rectum adenocarcinoma) datasets, respectively. Then, we performed an expression analysis of each CCL in different pathological stages through the “Single Gene Analysis” module, and a value of *p* ≤ 0.05 was considered credible.

### 4.4. UALCAN

UALCAN is a comprehensive, user-friendly, and interactive web resource for analyzing cancer OMICS data. We searched the UALCAN (http://ualcan.path.uab.edu/) platform, a comprehensive web resource based on data from The Cancer Genome Atlas (TCGA) [54], to obtain the co-expressed genes of the CCLs selected for research and explore the interactions between genes. In detail, the genes positively and/or negatively correlated with CCLs in COAD and READ analyzed by Pearson analysis were identified using the “TCGA analysis” module. The *p*-value cutoff was 0.05.

### 4.5. cBioPortal

cBioPortal (www.cbioportal.org), a visual tool for analyzing cancer genetic data [55], was visited to obtain information on CCLs’ genetic alteration in CRC and analyze these alterations to explore their impact on CRC development. First of all, 1510 CRC samples based on the TCGA database were selected for further analysis. Then, we inputted our CCLs and explored the “Oncoprint” module to confirm their mutation or amplification in each CRC sample.

### 4.6. HPA

The Human Protein Atlas is a database that maps all the human proteins in cells, tissues, and organs using various omics technologies. We drew Kaplan–Meier survival curves to characterize the potential prognostic value and evaluate CCL protein expression and its correlation with CRC patient outcomes of CCLs in CRC based on TCGA data downloaded from HPA (https://www.proteinatlas.org/) [56] using the SPSS 19.0 software package (SPSS Inc., Chicago, IL, USA), and the *p*-values were calculated by using log-rank tests with a cutoff of less than 0.05.

### 4.7. Gene MANIA and STRING

GeneMANIA (http://www.genemania.org) [57] and STRING (https://STRING-db.org/) [58] were used to predict the gene–gene interaction networks involving CCLs to identify signaling pathways in our study. Specifically, we analyzed the co-expression, co-localization, and protein domain similarity of the submitted CCLs and even other relevant proteins via Gene MANIA. Similarly, we conducted a further analysis to explore the interactions among the differentially expressed CCLs and their co-expressed mutant genes through STRING.

### 4.8. Shbio and Metascape

Shbio (http://enrich.shbio.com/) and Metascape (http://metascape.org) are visual tools for the enrichment analysis of multiple genes, which were used to further clarify and investigate the cellular functions of the selected CCLs and their co-expressed mutant genes [59]. Herein, the enrichment of CCLs and significantly co-expressed genes was verified by conducting the Kyoto Encyclopedia of Genes and Genomes (KEGG) pathway enrichment analysis and Gene Ontology (GO) enrichment analysis. Among them, GO analysis simultaneously provided a register of the biological processes (BPs), cellular components (CCs), and molecular functions (MFs) that target genes participated in, and the threshold value was *p* ≤ 0.05.

### 4.9. TRRUST and Linked Omics

We explored the upstream transcription factors (TFs) and downstream kinases of these CCLs using TRRUST (https://www.grnpedia.org/trrust/, accessed on 10 May 2022) [60] and Linked Omics (http://www.linkedomics.org/) [61], respectively. More specifically, TRRUST was used to find key regulators of these indicators for TFs often simultaneously regulating multiple genes whose functions are closely related. Herein, seven candidate genes, including CCL2/3/4/5/11/21/26, were effectively queried in the database. In addition, we created a new analysis based on RNA-seq data from TCGA-COAD READ, and Gene Set Enrichment Analysis (GSEA) for kinase targets was performed to identify CCL-mediated downstream genes via the “Link Interpreter” module. The following parameters were used to determine statistically significant differences: normalized enrichment score (NES) > 1, nominal (NOM) *p*-value < 0.05, and false discovery rate (FDR) < 25%.

### 4.10. TIMER

TIMER (https://cistrome.shinyapps.io/timer/, accessed on 13 May 2022) was used in this study for multivariate prognostic analysis based on the “Survival” module [62]. The variables selected in the Cox proportional hazard model were not only these CCLs but also immune infiltrates, and TIMER outputted the Cox regression results containing hazard ratios and statistical significance automatically. The coefficient reads as a regression coefficient. HR gives you the hazard ratio, and its lower and upper 95% confidential intervals are shown in 95%CI_l and 95%CI_u.

### 4.11. Sanger Box

Sanger Box (http://sangerbox.com/), a robust data analysis platform, facilitated our investigation into the correlations between CCL expression and six types of tumor infiltration-related immune cells. Additionally, it enabled the identification of gene intersections across multiple datasets using the “Gene+” and “Tool+” modules. Specifically, the immune cell scores for these CCLs in colorectal cancer (CRC) were calculated by selecting tumor types such as COAD and READ. Furthermore, CCL-related pathway proteins were identified by constructing a Venn diagram based on the co-expressed and mutated genes of CCLs in CRC.

## Figures and Tables

**Table 1:** The dysregulation of CCLs between CRC and normal tissues at transcriptome level based on the datasets from ONCOMINE.

Indicator	Type	Fold Change	*p*-Value	*t*-Test	Reference
CCL2	Rectal Adenoma	−3.318	2.82 × 10^−4^	−5.574	[17]
CCL3	Colorectal Carcinoma	3.319	4.30 × 10^−7^	5.774	[18]
CCL4	Colorectal Carcinoma	2.405	1.76 × 10^−6^	5.416	[18]
CCL5	Rectosigmoid Adenocarcinoma	−8.903	5.76 × 10^−4^	−9.798	[19]
CCL21	Rectosigmoid Adenocarcinoma	−7.038	9.50 × 10^−8^	−24.969	[20]
CCL26	Colon Mucinous Adenocarcinoma	5.276	1.15 × 10^−7^	6.791	[21]
CCL28	Colorectal Carcinoma	−5.073	1.22 × 10^−22^	−13.508	[16]

**Table 2:** The key regulatory factors of CCL transcription in CRC, as identified by the TRRUST database.

Key TF	Description	Regulated Genes	*p*-Value	FDR
RELA	v-rel reticuloendotheliosis viral oncogene homolog A (avian)	CCL4, CCL2, CCL3, CCL11, CCL5	5.37 × 10^−8^	2.78 × 10^−7^
NFKB1	nuclear factor of kappa light polypeptide gene enhancer in B cells 1	CCL5, CCL3, CCL11, CCL4, CCL2	5.55 × 10^−8^	2.78 × 10^−7^
IRF3	interferon regulatory factor 3	CCL2, CCL5	1.65 × 10^−5^	5.49 × 10^−5^
REL	v-rel reticuloendotheliosis viral oncogene homolog (avian)	CCL2, CCL5	3.62 × 10^−5^	9.05 × 10^−5^
STAT6	signal transducer and activator of transcription 6, interleukin-4 induced	CCL11, CCL26	9.84 × 10^−5^	0.000197
SPI1	spleen focus-forming virus (SFFV) proviral integration oncogene spi1	CCL2, CCL5	0.000294	0.000489
STAT1	signal transducer and activator of transcription 1, 91 kDa	CCL2, CCL3	0.000539	0.00077
STAT3	signal transducer and activator of transcription 3 (acute-phase response factor)	CCL11, CCL2	0.00153	0.00187
JUN	jun proto-oncogene	CCL5, CCL2	0.00168	0.00187
SP1	Sp1 transcription factor	CCL2, CCL5	0.0158	0.0158

**Table 3:** The kinase target networks of CCLs in CRC.

CL Chemokines	Enriched Kinase Target	Description	NES	*p*-Value	FDR
CCL2	Kinase_HCK	HCK proto-oncogene, Src family tyrosine kinase	1.38	0.002	0.15
	Kinase_PIK3CD	phosphatidylinositol-4,5-bisphosphate 3-kinase catalytic subunit delta	1.40	0.002	0.16
CCL3	Kinase_LYN	LYN proto-oncogene, Src family tyrosine kinase	1.66	0	0
	Kinase_HCK	HCK proto-oncogene, Src family tyrosine kinase	1.65	0	0
CCL4	Kinase_LCK	LCK proto-oncogene, Src family tyrosine kinase	1.70	0	0
	Kinase_LYN	LYN proto-oncogene, Src family tyrosine kinase	1.69	0	0
CCL5	Kinase_LYN	LYN proto-oncogene, Src family tyrosine kinase	1.77	0	0
	Kinase_LCK	LCK proto-oncogene, Src family tyrosine kinase	1.75	0	0
CCL11	Kinase_LYN	LYN proto-oncogene, Src family tyrosine kinase	1.50	0	0.12
	Kinase_CSNK1G2	casein kinase 1 gamma 2	1.48	0	0.12
CCL21	Kinase_NEK2	NIMA-related kinase 2	−2.03	0	0.01
	Kinase_CSNK1G2	casein kinase 1 gamma 2	1.42	0	0.22
CCL26	Kinase_LYN	LYN proto-oncogene, Src family tyrosine kinase	1.84	0	0
	Kinase_ATR	ATR serine/threonine kinase	−1.89	0	0.05
CCL28	Kinase_ATM	ATM serine/threonine kinase	−1.79	0	0.06
	Kinase_CDK2	Cyclin-dependent kinase 2	−1.71	0	0.09

**Table 4:** Multivariate COX regression analysis of CCLs (CCL2/4/11/28) and six tumor-infiltrating cells.

Variables	Coef	HR (95% CI)	*p*-Value
B lymphocyte	1.381	3.977 (0.057–279.105)	0.524
CD8+ T cell	−4.142	0.016 (0–0.811)	**0.039**
CD4-positive cells	−0.479	0.619 (0.005–72.553)	0.844
Macrophage	1.904	6.713 (0.069–651.549)	0.415
Neutrophil	1.375	3.954 (0.001–14,434.802)	0.743
Dendritic cell	1.625	5.077 (0.28–92.048)	0.272
CCL2	0.247	1.28 (0.97–1.69)	0.081
CCL4	−0.262	0.77 (0.588–1.008)	0.057
CCL11	−0.23	0.795 (0.643–0.982)	**0.033**
CCL28	−0.07	0.932 (0.79–1.099)	0.403

## Data Availability

The original contributions presented in this study are included in the article. Further inquiries can be directed to the corresponding author.
